# Synergistic effects of ceftriaxone combined with BLI-489 against NDM-producing *Klebsiella pneumoniae*

**DOI:** 10.1128/spectrum.01575-25

**Published:** 2026-02-24

**Authors:** Nathalia da Silva Damaceno, Nathalia Monteiro Lins Freire, Mariana Carvalho Sturaro, Gleyce Hellen de Almeida de Souza, Izadora Dillis Faccin, Ana Cristina Gales, Thiago Mendonça de Aquino, Simone Simionatto

**Affiliations:** 1 Federal University of Grande Dourados (UFGD), Research in Health Science Laboratory, Dourados, Brazil; 2 Federal University of Alagoas (UFAL), Research Group on Therapeutic Strategies, Institute of Chemistry and Biotechnology 425903, Maceió, Brazil; 3 Division of Infectious Diseases, Department of Internal Medicine, Federal University of São Paulo (UNIFESP), Laboratório Alerta, Paulista School of Medicine, São Paulo, São Paulo, Brazil; 4 Antimicrobial Resistance Institute of São Paulo, São Paulo, Brazil; Universita degli Studi dell'Insubria, Varese, Italy

**Keywords:** ceftriaxone, BLI-489, NDM-*Klebsiella pneumoniae*, combination therapy

## Abstract

The New Delhi metallo-β-lactamase-producing *Klebsiella pneumoniae* (NDM-Kp) strain is a high-risk pathogen responsible for many multidrug-resistant (MDR) infections worldwide, particularly in healthcare settings. The scarcity of effective metallo-β-lactamase (MBL) inhibitors highlights the urgent need for novel therapeutic strategies to combat this threat. In this study, we evaluated the synergistic effects of ceftriaxone (CRO) with the β-lactamase inhibitor BLI-489, aiming to restore the efficacy of cephalosporins against NDM-*Kp* strains. The CRO/BLI-489 exhibited significant synergistic activity *in vitro*, as indicated by a fractional inhibitory concentration index (FICI) of 0.25. The combination significantly reduced the minimum inhibitory concentration (MIC) and minimum bactericidal concentration (MBC) against NDM-*Kp*. This combination also inhibited biofilm formation. Hemolysis assays revealed that the hemolytic activity was significantly lower after treatment with CRO/BLI-489 compared to that recorded after treatment with each compound alone. This finding indicated that the combination treatment has higher biocompatibility and retains antimicrobial efficacy. Molecular dynamics (MD) simulations revealed that the synergistic antibacterial effect of the CRO/BLI-489 is mediated by interactions involving coordination with Zn^2+^ ions in the catalytic site of the MBL enzyme. These results suggest that the combination can overcome MBL-mediated resistance mechanisms.

**IMPORTANCE:**

The global spread of NDM-Kp poses a major challenge to healthcare systems because of the scarcity of effective treatment options. In response to the urgent need for new therapeutic strategies, this study explores the CRO/BLI-489 as a promising alternative for overcoming MBL-mediated resistance. Although not yet an established therapy, this approach demonstrates the potential to restore cephalosporin efficacy, inhibit biofilm formation, and reduce cytotoxicity. These results offer valuable insights that may guide the development of future treatments for infections caused by multidrug-resistant pathogens. Our findings underscore the importance of further preclinical and clinical studies to validate this strategy and contribute to the global effort against antibiotic resistance.

## INTRODUCTION

The global rise of antimicrobial resistance (AMR) poses a serious threat to public health. Carbapenem-resistant *Klebsiella pneumoniae* has been classified as a critical-priority pathogen by the World Health Organization, underscoring the urgent need for the development of novel therapeutic strategies (1, 2). The primary mechanism underlying carbapenem resistance in *K. pneumoniae* and other *Enterobacterales* is the production of carbapenemases. Historically, *Klebsiella pneumoniae* carbapenemase (KPC) was the most prevalent carbapenemase identified in Latin America (LATAM). However, recent data from the ATLAS surveillance program have documented the emergence of New Delhi metallo-β-lactamase (NDM) as the predominant carbapenemase, not only in LATAM but also across the Asia-Pacific and Middle East-Africa regions (3).

The clinical threat posed by NDM-producing *Klebsiella pneumoniae* (NDM-*Kp*) is primarily associated with three key characteristics: (i) resistance to all or nearly all available antibiotics, including last-line agents, such as carbapenems; (ii) horizontal transfer of resistance determinant, *bla*_NDM_, to other bacteria (e.g., *Escherichia coli*) via plasmid-mediated conjugation; and (iii) a high propensity for dissemination in healthcare environments (4–6). NDM-*Kp* infections are further exacerbated by the organism’s robust biofilm-forming capacity and the frequent co-occurrence of multiple resistance genes, which contribute to persistent infections and limited treatment efficacy, particularly in nosocomial settings (7–9).

The combination of ceftazidime-avibactam with aztreonam has been recommended for the management of infections caused by MBL-producing *Enterobacterales* (10). This strategy is based on the stability of aztreonam against MBL-mediated hydrolysis, coupled with avibactam’s ability to inhibit most other clinically relevant β-lactamases. Nevertheless, the synergistic efficacy of this combination requires confirmation prior to clinical use due to the potential absence of synergy, which may compromise therapeutic effectiveness (11). Other therapeutic options for infections caused by MBL-producing organisms are largely limited to non-β-lactam agents, such as polymyxins, aminoglycosides, tigecycline, and fosfomycin; however, their clinical utility is constrained by factors, including nephrotoxicity, hepatotoxicity, and suboptimal pharmacokinetic/pharmacodynamic profiles (10, 12).

Aztreonam-avibactam has demonstrated potent *in vitro* activity against metallo-β-lactamase (MBL)-producing *Enterobacterales* and recently received regulatory approval for clinical use by both the European Medicines Agency and the U.S. Food and Drug Administration. In the European Union and the United Kingdom, aztreonam-avibactam is approved for a broader range of indications, including hospital-acquired pneumonia, ventilator-associated pneumonia, complicated urinary tract infections, and complicated intra-abdominal infections (cIAI). Conversely, in the United States, aztreonam-avibactam has been approved solely for the treatment of cIAI in adult patients with limited or no alternative therapeutic options. This restricted approval is attributable to the fact that, although the pivotal clinical trial was conducted in regions with a high prevalence of MBL-producing gram-negative pathogens, only a limited number of enrolled patients were infected with MBL-producing organisms. This underscores the necessity for additional clinical data to robustly establish the efficacy of aztreonam-avibactam against such pathogens (13). Furthermore, the emergence of resistance to aztreonam-avibactam has already been reported, primarily associated with structural alterations in PBP3, in *E. coli*, and increased *bla*_KPC_ copy number, porin loss (OmpK35/OmpK36), and efflux pump overexpression in *K. pneumoniae*, highlighting the need for continued surveillance and the development of complementary therapeutic strategies (14, 15).

Despite the increase in antibiotic development over the past decade, the absence of clinically approved inhibitors specifically targeting MBLs underscores the urgent need to develop alternative therapeutic strategies (5, 16–18). The structural modification of a penem inhibitor through the introduction of a heterocyclic moiety at position 6 via a methylene linkage significantly enhanced its activity against a broad spectrum of β-lactamases. Among promising candidates, BLI-489, a bicyclic 6-methylidene penem compound, has exhibited potent inhibitory activity against Amber class A, C, and D β-lactamase-producing pathogens when combined with piperacillin (19–25). Although not yet approved for clinical use, its potential utility within combination regimens warrants further investigation.

In this study, we aimed to evaluate the activity of the ceftriaxone (CRO)/BLI-489 combination against NDM-*Kp* by assessing its ability to restore the β-lactam activity, reduce cytotoxicity, and inhibit biofilm formation, contributing to provide key preclinical insights into a potential strategy for managing infections caused by NDM-*Kp* and other multidrug-resistant pathogens.

## RESULTS

**Fig 1:**
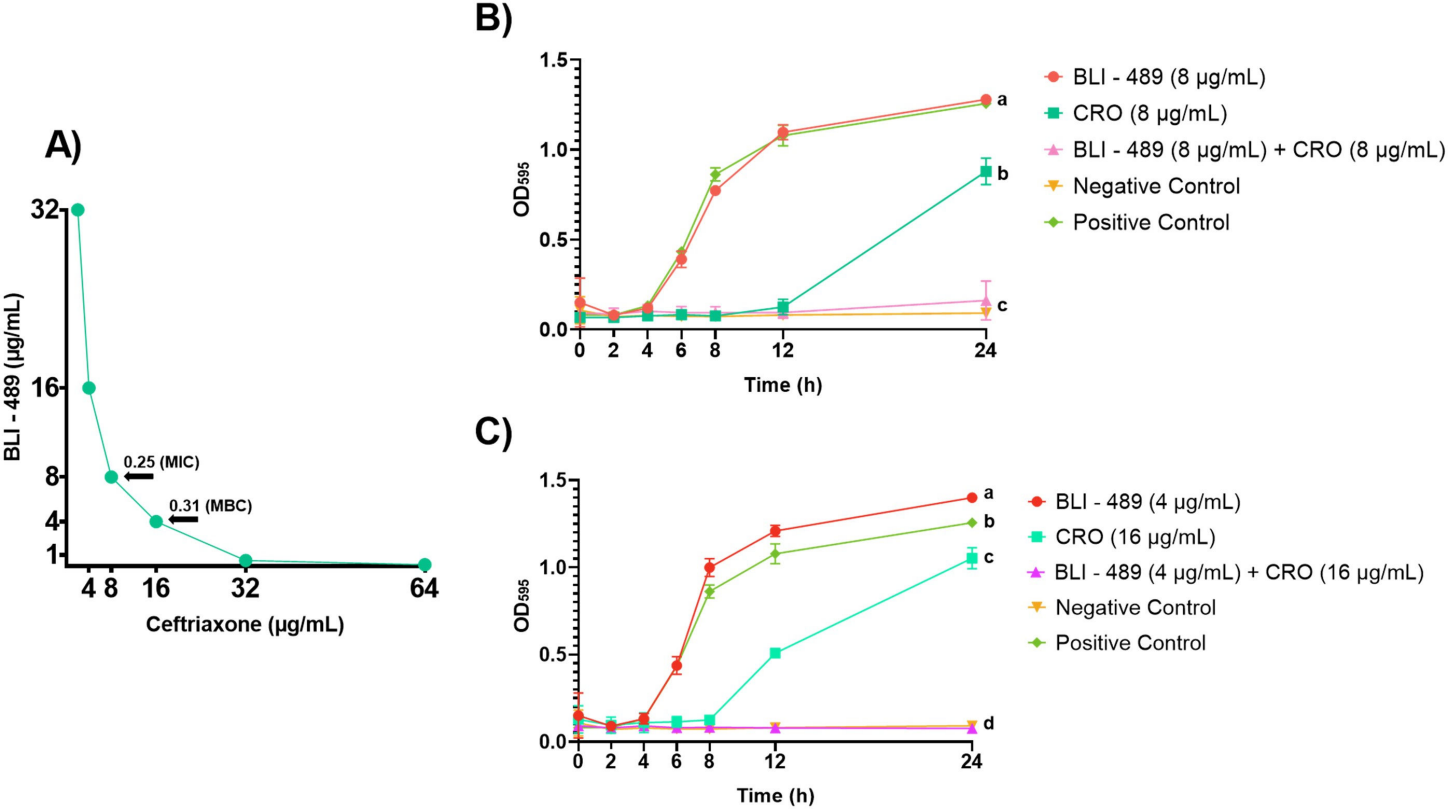
Synergistic effect of the CRO/BLI-489 combination against NDM-*Kp*. (**A**) CRO/BLI-489 concentrations that inhibited bacterial growth are shown. The arrows indicate the fractional inhibitory concentration index (FICI) corresponding to the MIC (FICI = 0.25) and MBC (FICI = 0.31). (**B**) Growth curve for the CRO/BLI-489 combination at the MIC is illustrated. Unlike the isolated antibiotics, which formed a curve similar to that of the positive control, the combination treatment resulted in a linear growth trend. (**C**) Growth curve for CRO/BLI-489 at the MBC is shown. The combination established bacterial growth, whereas the isolated compounds did not, resulting in a curve similar to that of the positive control. The positive and negative controls correspond to the bacterial suspensions in broth and sterile broth, respectively. Tukey’s test at 99% confidence was performed, and treatments with the same letter indicate statistically similar means. MIC, minimal inhibitory concentration; MBC, minimal bactericidal concentration; CRO, ceftriaxone.

The MIC of BLI-489 and CRO alone was >64 µg/mL. The CRO/BLI-489 exhibited synergistic interactions *in vitro*. The MBC was determined at 16 µg/mL CRO and 4 µg/mL BLI-489, which indicated that the CRO/BLI-489 had a synergistic effect (Table 1). The CRO/BLI-489 inhibited NDM-*Kp* growth at various associated concentrations (Fig. 1A). Synergistic effects were confirmed by low FICI values of 0.25 at bacteriostatic concentrations and 0.31 at bactericidal concentrations. Additionally, CRO/BLI-489 shows the potential to establish NDM-*Kp* microbial growth within the MIC and MBC concentrations, as well as the negative control does, according to Tukey’s test with a 99% confidence level. In contrast, the isolated compounds could not replicate the desired outcome. BLI-489 exhibited microbial growth patterns comparable to those of the positive control, while the inhibitory effects of CRO persisted for only 12 h before regrowth occurred (Fig. 1B and C).

**TABLE 1:** Antibacterial results for CRO and BLI-489^a^

Parameter	Isolated CRO MIC	Isolated BLI-489 MIC	CRO	BLI-489	FICI	Interaction
MIC	>64	>64	8	8	0.25	Synergic
MBC	>64	>64	16	4	0.31	Synergic

^a^ MIC and MBC values are represented as µg/mL. MIC, minimal inhibitory concentration; MBC, minimal bactericidal concentration; CRO, ceftriaxone.

**Fig 2:**
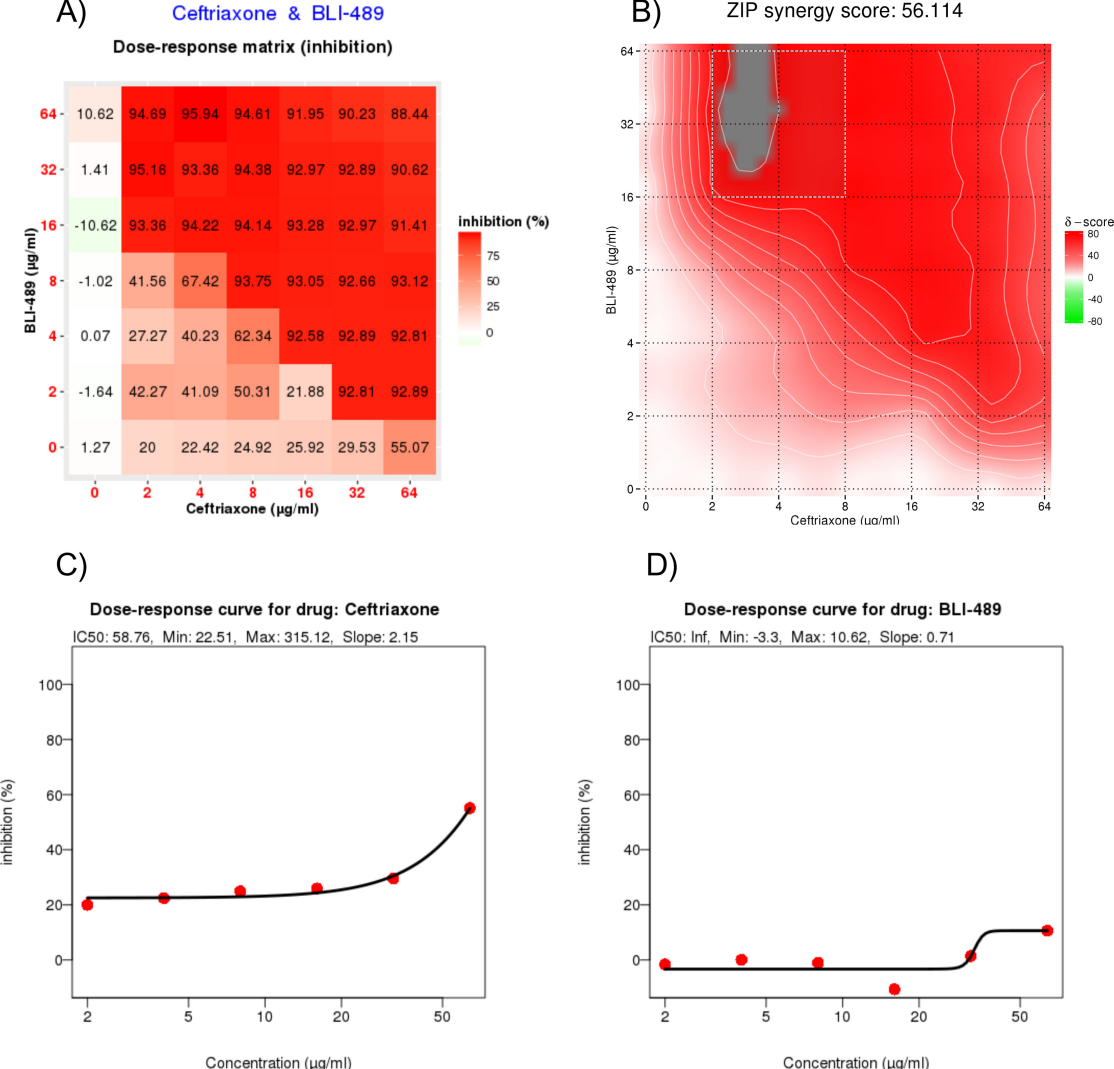
SynergyFinder analyses. (**A**) Dose-response matrix of the CRO/BLI-489 association is shown. The reddest areas identified the dose combinations that inhibited >80% of bacterial growth. (**B**) Hot zone for the CRO/BLI-489 combination is shown; the ZIP synergy score of 56.114 indicates synergism between the compounds. (**C**) Dose-response curve for CRO. (**D**) Dose-response curve for BLI-489.

To further investigate the synergistic interaction between CRO and BLI-489, a comprehensive SynergyFinder analysis was performed. The results of the analysis revealed a strong synergy, with the CRO/BLI-489 exhibiting a robust ZIP synergy score of 56.114, which indicated potent cooperative effects. Microbial inhibition was observed within a concentration range of 16–64 µg/mL BLI-489 associated with 2–8 µg/mL CRO (Fig. 2A and B). The inhibition percentages scaled proportionally with increasing concentrations of the CRO/BLI489, reinforcing the dose-dependent antimicrobial efficacy (Fig. 2C and D).

**Fig 3:**
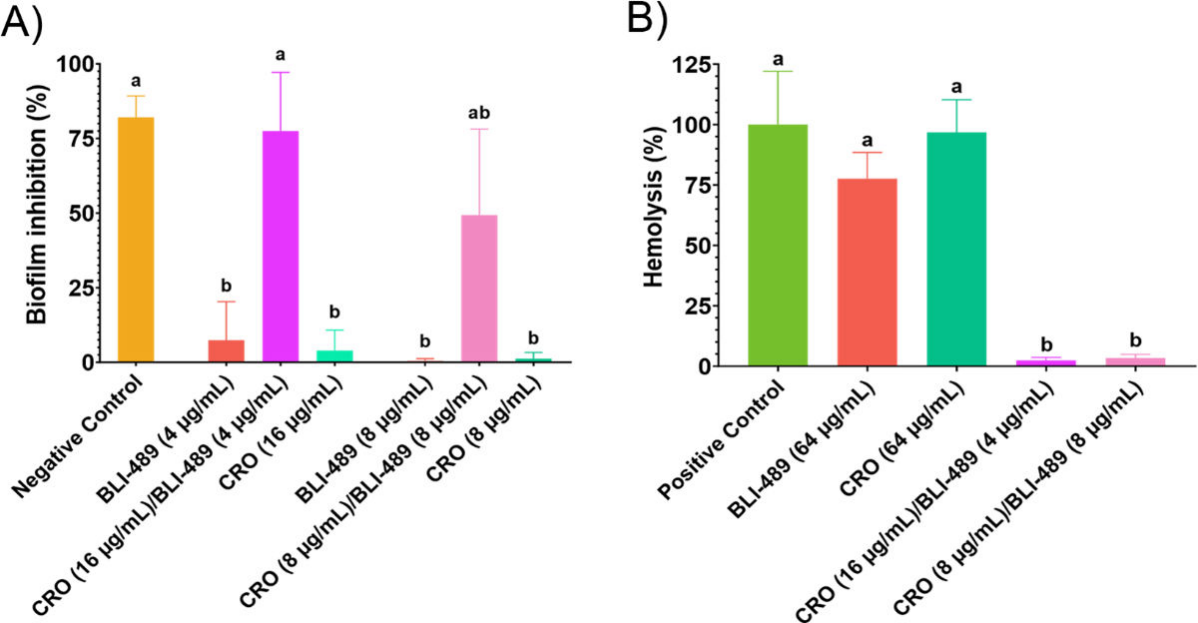
Biofilm formation inhibition and hemolytic activity results. (**A**) Biofilm inhibition % is shown for CRO/BLI-489 combinations and isolated drugs for MIC (8 µg/mL each) and MBC (16 µg/mL CRO + 4 µg/mL BLI-489). The negative control corresponds to sterile broth. (**B**) Hemolysis % is shown for CRO/BLI-489 combinations: MIC and MBC and isolated drugs at their MIC (64 µg/mL). Triton X-100 (0.1%, vol/vol) was used as a positive control. Tukey’s test at 99% confidence was performed, and treatments with the same letter indicate statistically similar mean values. MIC, minimal inhibitory concentration; MBC, minimal bactericidal concentration; CRO, ceftriaxone.

The CRO/BLI-489 also exhibited biofilm inhibition potential (Fig. 3A). BLI-489 at 8 and 16 µg/mL had OD values of 0.727 ± 0.294 and 0.522 ± 0.169, respectively, corresponding to biofilm inhibition rates of approximately 0.456 and 7.44%, respectively. Similarly, CRO at 8 and 4 µg/mL had OD values of 0.500 ± 0.077 and 0.561 ± 0.254, respectively, indicating biofilm inhibition rates of 1.21 and 3.95%, respectively. The CRO/BLI-489 MIC yielded an OD of 0.223 ± 0.127, corresponding to 49.32% biofilm inhibition, whereas the MBC further reduced the OD to 0.099 ± 0.086, resulting in 77.52% inhibition. Both combinations presented statistically similar means to those of the negative control (*P* < 0.01).

To assess the cytotoxicity and hemocompatibility, a hemolysis assay was conducted to determine the ability of the CRO/BLI-489 to lyse red blood cells (RBCs). For comparison, the individual drugs were tested at their MIC (64 µg/mL). BLI-489 and CRO exhibited hemolysis percentages of 77.56 and 96.75% with corresponding OD values of 0.588 ± 0.078 and 0.726 ± 0.097, respectively, which were statistically similar to those of the positive control (*P* < 0.01). In contrast, the CRO/BLI-489 at both the MIC and MBC concentrations exhibited statistically significant differences compared to the positive control (*P* < 0.01), with low hemolysis percentages of 3.35 and 2.37% and ODs of 0.056 ± 0.011 and 0.049 ± 0.009, respectively (Fig. 3B).

**Fig 4:**
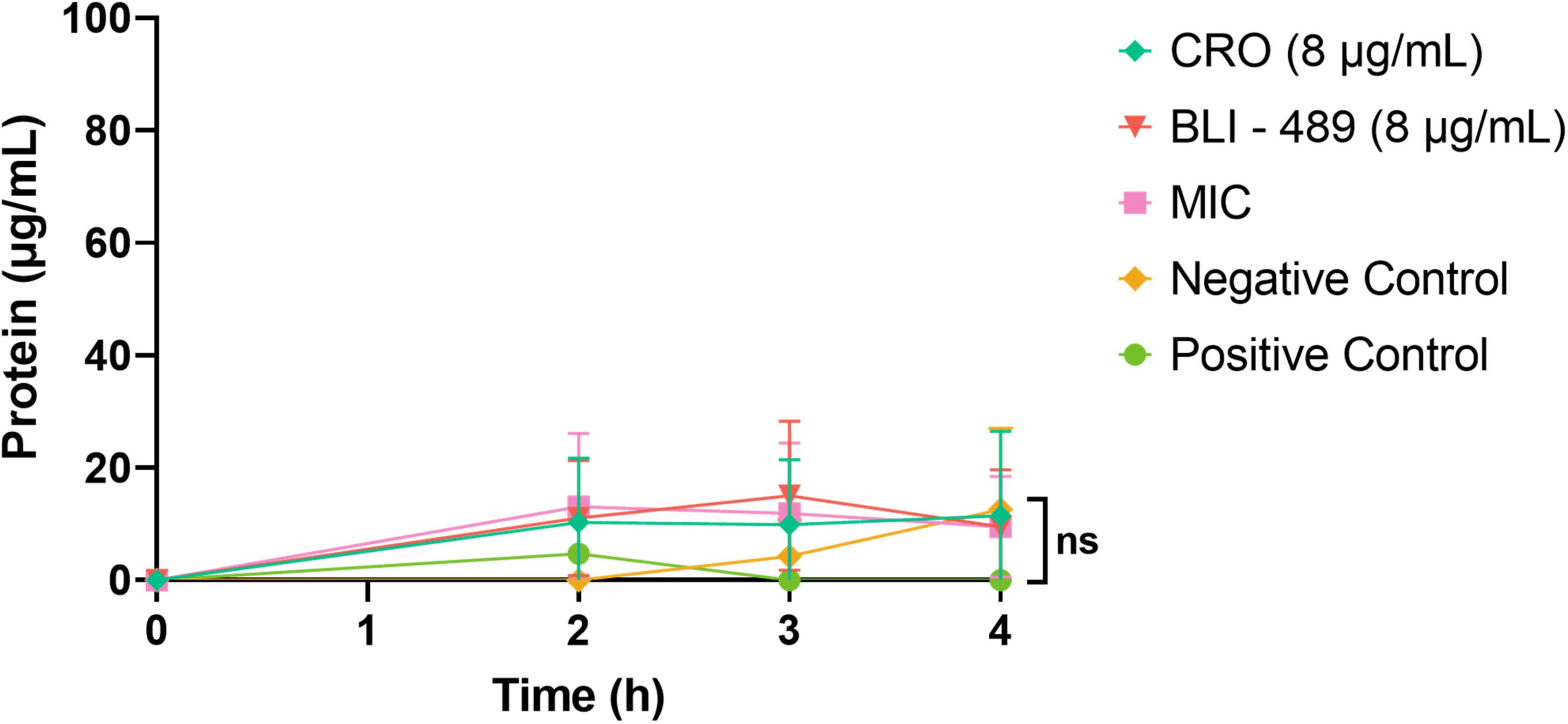
Protein leakage activity was analyzed using Dunnett’s multiple-comparisons test. Protein concentrations were measured for the CRO/BLI-489 MIC combination (8 µg/mL each) and the individual drugs. No significant protein leakage was observed. MIC: minimal inhibitory concentration, CRO: ceftriaxone, negative control: sterile water, and positive control: bacteria in sterile water.

**Fig 5:**
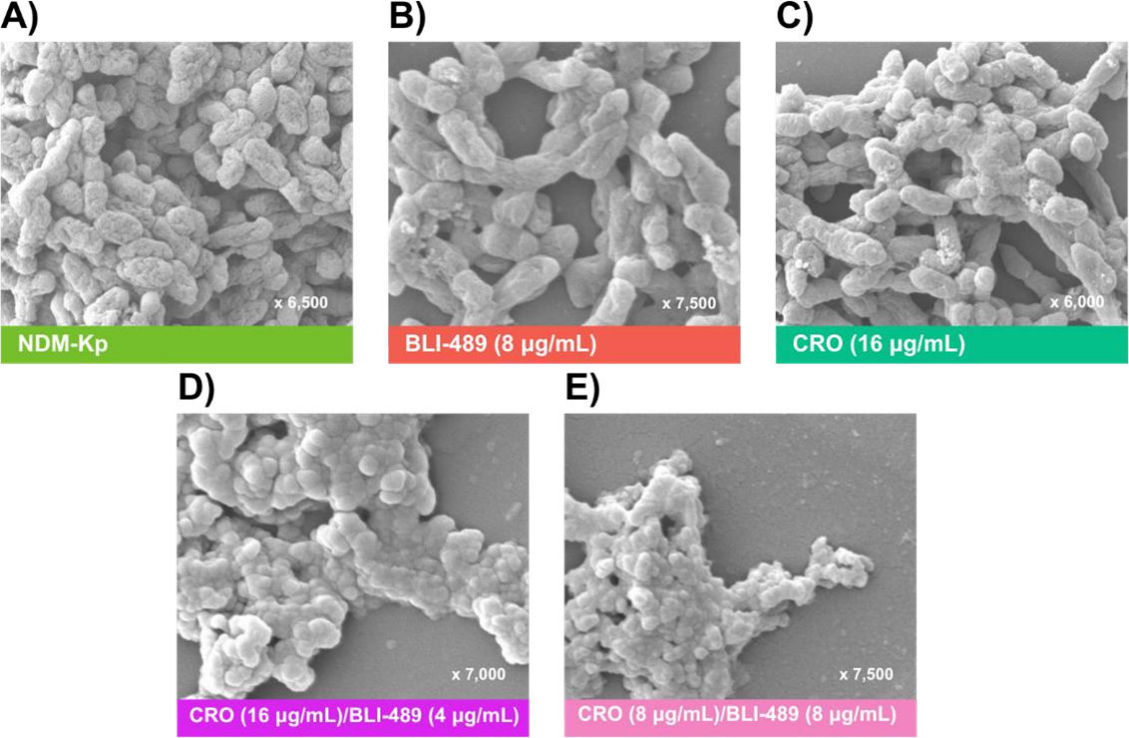
The SEM images show the cell morphology. (**A**) Untreated bacterial strain NDM-*Kp*. (**B**) Bacteria treated with BLI-489 alone (8 µg/mL). (**C**) Bacteria treated with CRO alone (16 µg/mL). (**D**) Bacteria treated with CRO/BLI-489 at the MIC (8 µg/mL each). (**E**) Bacteria treated with CRO/BLI-489 at MBC concentration (16 µg/mL CRO + 4 µg/mL BLI-489). MIC: minimal inhibitory concentration, MBC: minimal bactericidal concentration, and CRO: ceftriaxone.

Cell membrane permeability and SEM analyses were conducted to assess the effect of CRO/BLI-489 on NDM-*Kp* cell integrity. The membrane permeability assay indicated that CRO/BLI-489 did not cause significant protein leakage, suggesting that membrane disruption is not the primary mechanism of action (Fig. 4). The SEM images revealed that the bacterial load was lower after treatment with the CRO/BLI-489 compared to that recorded after treatment with the isolated compounds and the NDM-*Kp* control. This finding highlighted the efficacy of the combination in reducing the number of structurally preserved bacteria. Moreover, no surface damage was observed in bacterial morphology when the bacteria were treated with either bacteriostatic or bactericidal concentrations of CRO/BLI-489 (Fig. 5).

The *in silico* study was conducted to characterize the interactions between BLI-489 and MBLs produced by NDM-*Kp*. The findings provided insights into the activity of BLI-489 in combination therapy. Initially, for docking protocol validation and selection of the optimal PDB entry and scoring function, a redocking experiment was conducted, where each co-crystallized ligand was extracted from 25 original PDB entries into their corresponding target structure using all four scoring functions available in Gold 2020. PDB entry 4EY2 was selected because it presented the lowest average RMSD across all tested PDB entries (1.1064). Additionally, the scoring function ChemPLP was selected because of its superior performance, yielding the lowest RMSD value (0.060) for the selected PDB entry.

**Fig 6:**
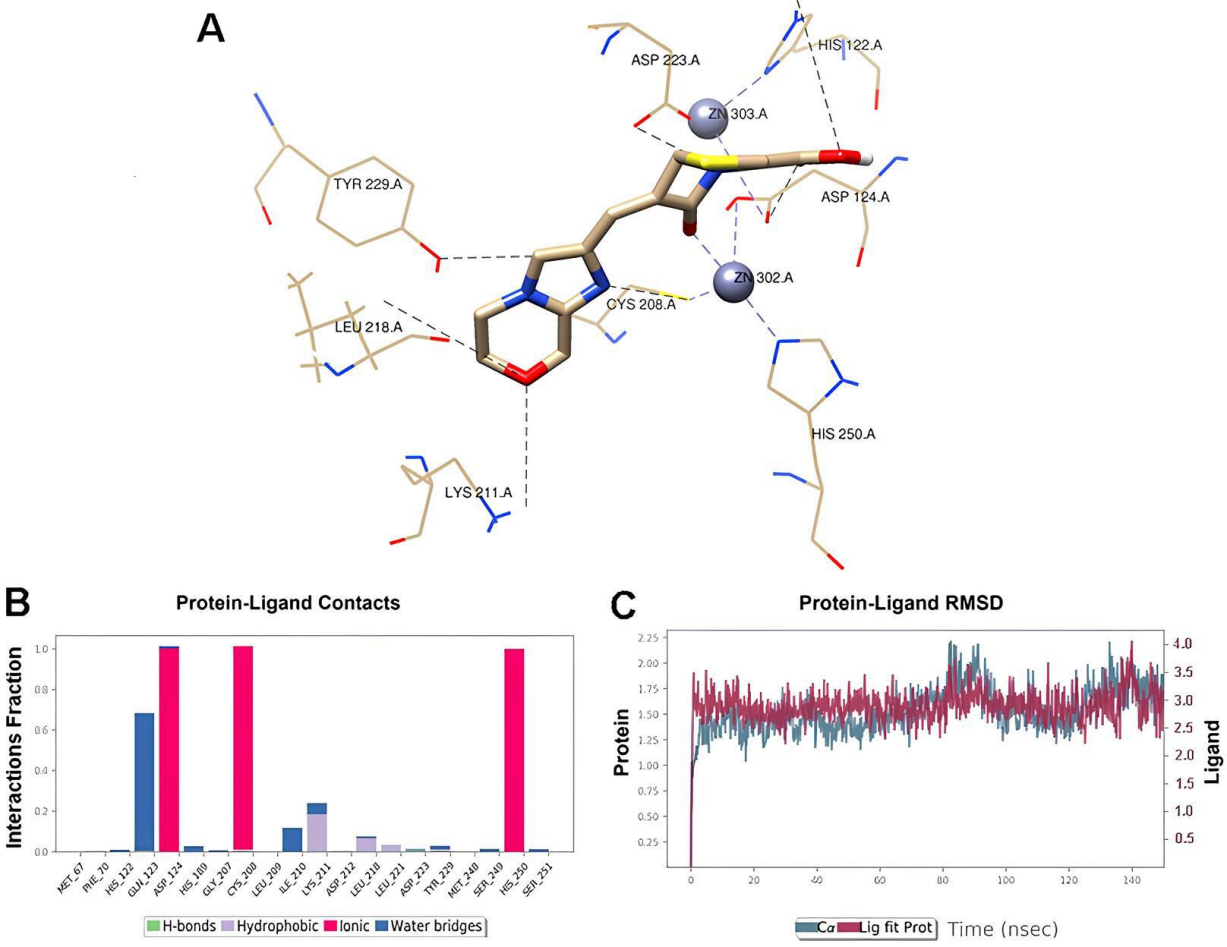
Molecular docking and dynamic simulations. (**A**) Protein interactions with BLI-489 were monitored throughout the MD simulation. A value of 1.0 suggests that the specific interaction is sustained throughout the simulation, whereas values greater than 1.0 occur when the residue establishes multiple contacts of the identical subtype with the ligand. (**B**) A 3D representation of the interactions between BLI-489 and MBL. The binding conformation of the ligand is visualized using a stick representation, where the green spheres denote the two Zn metals. The coordination with metals, water bridges, and hydrophobic and ionic interactions is illustrated using dotted lines. (**C**) RMSD plots of the MBL backbone and BLI-489 in the catalytic cavity of jack bean urease are shown.

Docking simulations were conducted for BLI-489, and the lowest-energy binding pose was used as the initial conformation for 150 ns MD simulations. The MD results revealed that the oxygen atoms of the β-lactam carbonyl and carboxylic acid groups played an important role in coordinating with Zn^2+^ ions, forming a stable ligand-enzyme complex crucial for activity (Fig. 6A). Throughout the simulation, the complex was stabilized by ionic interactions with the Cys208, His250, and Asp124 residues in the catalytic site, with the latter being essential for the MBL activity (Fig. 6B). Finally, the RMSD ranged from 1.0 to 2.5 Å, strongly suggesting stability within the catalytic site during all MD simulations. The RMSD plot for the BLI-489 complex is shown in Fig. 5C.

## DISCUSSION

The spread of NDM-*Kp* strains is a serious threat to public health. In 2019, 22.2% of clinical *K. pneumoniae* isolates worldwide harbored NDM-type enzymes (16). Outbreaks involving NDM-*Kp* have been reported across several countries, including Australia, China, Iran, France, Poland, South Africa, and the United States (26–32). The scarcity of clinically approved MBL inhibitors significantly limits therapeutic options (33), highlighting the urgent need for novel inhibitors and treatment strategies to combat NDM-*Kp* and mitigate its global health effects.

Ceftriaxone is a widely used, cost-effective broad-spectrum β-lactam with activity against gram-positive and -negative pathogens, especially *Enterobacteriaceae* (34). Its once-daily administration further reduces labor and treatment costs, contributing to its extensive use for infections, such as pneumonia and urinary tract infections, especially in low- and middle-income countries (35–37). Nevertheless, the widespread reliance has been accompanied by a growing prevalence of ceftriaxone resistance (35). Thus, identifying strategies to restore its activity against high-priority pathogens, including NDM-*Kp*, is critical.

The combination of β-lactam antibiotics with BLIs is a promising strategy to restore antimicrobial activity against β-lactamase-producing bacteria. BLIs, such as avibactam and vaborbactam, restore cephalosporin activity by inhibiting key resistance enzymes, providing effective options for treating MDR infections. Consistent with these findings, our checkerboard assay demonstrated synergistic interactions between CRO and BLI-489, yielding a low FICI of 0.25 (synergy). Drug combinations can yield antagonistic, indifferent, or synergistic interactions, and the checkerboard assay together with FICI, a validated and widely accepted metric, provides a fundamental framework for their systematic *in vitro* evaluation. The CRO/BLI-489 inhibited the growth of NDM-*Kp* at concentrations significantly lower than those required when the antibiotics were tested individually, indicating synergism.

This study expands upon previous findings by demonstrating the biofilm-inhibitory capacity of CRO/BLI-489, a property likely attributable to its enhanced antibacterial activity. Biofilm formation is a key virulence factor in *K. pneumoniae* and other carbapenemase-producing organisms that significantly contributes to AMR (8). Biofilms can be found not only on the skin, mucosa, and teeth of humans, but also in implantable medical devices, such as central venous catheters or artificial hip or knee joints, thereby challenging treatment and resulting in treatment failure (38, 39). These findings highlight the potential of BLI-489 in mitigating persistent infections where biofilms play a crucial role, such as hospital-acquired infections.

Hemolysis assays were performed to assess the hemocompatibility of the CRO/BLI489. When tested individually at the higher concentrations required for bacterial inhibition, the compounds showed a relatively elevated hemolytic activity. In contrast, their effective concentrations in combination were markedly lower, resulting in hemolysis levels proportional to this reduced exposure. The CRO/BLI-489 combination showed hemolysis well below the 5% acceptability threshold, indicating improved biocompatibility and supporting a therapeutic advantage by minimizing cytotoxicity while preserving antimicrobial activity (40). This aligns with the known low hemolytic potential of β-lactams at clinically relevant concentrations (41).

Given the importance of MBL-mediated resistance, molecular dynamic (MD) simulations were conducted to elucidate the molecular interactions underlying the activity of the CRO/BLI-489. The simulations revealed that BLI-489 interacts directly with Zn^2+^ ions in the catalytic site of MBL. MBLs, including those produced by NDM-*Kp* strains, require zinc for their hydrolytic activity. This has led to the identification of selective MBL inhibitors that target zinc-binding and -chelation mechanisms, aiming to restore susceptibility to β-lactam antibiotics (42–44). Our results suggest that the antibacterial mechanism of the CRO/BLI-489 involves coordination with Zn^2+^ ions within the MBL catalytic site, which agrees with strategies aimed at overcoming MBL-mediated resistance. However, additional studies, including enzymatic assays employing purified NDM protein, are needed to further elucidate and confirm proposed mechanism of action.

Although β-lactam/BLI combinations have been explored, little is known about strategies capable of restoring ceftriaxone activity against *NDM-Kp*, and data on penem-derived inhibitors targeting MBL-mediated resistance remain scarce (21–25, 33, 45). Our findings address this gap by demonstrating that BLI-489 can re-establish ceftriaxone activity against NDM-*Kp* while also reducing biofilm formation and reducing cytotoxicity. This expands current knowledge beyond existing regimens, such as aztreonam-avibactam (11, 12, 14), by providing experimental support for an alternative, cost-effective β-lactam backbone frequently used in LMICs (36, 37). These results have implications for antimicrobial stewardship and may inform surveillance and the development of next-generation MBL inhibitors.

### Conclusion

This study provides novel insights into the potential of BLI-489 as an effective adjuvant to CRO. These results expand the current understanding of β-lactam/β-lactamase inhibitor strategies against MBL-mediated resistance and justify further investigation of this combination. Future work should include kinetic and pharmacodynamic analyses, evaluation across a broader diversity of clinical isolates, and *in vivo* validation to determine the translational applicability of CRO/BLI-489 for the treatment of MDR gram-negative infections.

## MATERIALS AND METHODS

### Chemicals

Ceftriaxone (CRO) disodium (lot no. 96260058; Laboratório Teuto Brasileiro S.A., Anápolis, Brazil) was reconstituted, and a solution was prepared following the manufacturer’s instructions, whereas BLI-489, synthesized at the Federal University of Alagoas (UFAL) was reconstituted in dimethyl sulfoxide (DMSO), ensuring that its final concentration in the treatments did not exceed 1%.

### Microorganisms and cultivation conditions

A previously identified and characterized NDM-*Kp* clinical isolate was used in this study. The isolate was initially cultured in brain heart infusion (BHI) broth at 37°C for 24 h. After incubation, the culture was streaked onto BHI agar and incubated under the same conditions.

### Bacterial strain

The strain exhibited resistance to amoxicillin, aztreonam, colistin, imipenem, meropenem, and tigecycline (Table 2). For the experiments, the bacterial suspension was standardized to a turbidity equivalent to 0.5 McFarland (approximately 1.5 × 10^8^ colony forming units [CFU]/mL) using spectrophotometry corresponding to an optical density (OD) between 0.08 and 0.13. The bacterial suspension was subsequently diluted 1:100 (vol/vol) in BHI broth to achieve a final inoculum of 1.55 × 10^6^ CFU/mL (46, 47).

**TABLE 2:** Susceptibility profile of the NDM-*Kp* isolate to tested antibiotics

Antibiotic	Disc content (µg)	Zone of inhibition (mm)	CLSI interpretation^*a*^
Aztreonam	30	13	R
Colistin	10	0	–
Imipenem	10	16	R
Meropenem	10	14	R
Tigecycline	15	0	–

^*a*^ R, resistant; I, intermediate; S, susceptible; –, there are no CLSI clinical breakpoints for defining the susceptibility category.

### Antibacterial assay

The antimicrobial activities of CRO and BLI-489 against the NDM-*Kp* strain were determined using a microdilution method following the Clinical and Laboratory Standards Institute (CLSI) guidelines (47, 48). Briefly, serial dilutions of each compound were prepared in 96-well polystyrene microtiter plates, with concentrations ranging from 0.125 to 64 µg/mL. After the bacterial suspension (1.5 × 10^6^ CFU/mL) was added to each well, the plates were incubated at 37°C for 24 h. Positive (NDM-*Kp* without antibiotics to assess cell viability) and negative (BHI broth to assess sterility) controls were used. The minimum inhibitory concentration (MIC) was considered the minimum dosage that inhibited visible microbial growth. To determine the minimum bactericidal concentration (MBC), aliquots from wells showing no visible growth were plated on BHI agar, and MBC was considered the minimum concentration with no growth of NDM-*Kp* (49).

### Checkerboard assay

A checkerboard assay was performed to investigate the interaction between BLI-489 and CRO (named CRO/BLI-489). Double serial dilutions of BLI-489 and CRO were dispensed in horizontal and vertical lines in a microtiter plate. Both antimicrobial agents were cross-diluted, and a standardized inoculum was used. The plates were incubated at 37°C under stationary conditions for approximately 24 h. As a positive control, the bacterial suspension in broth was used to evaluate growth, and as a negative control, broth was tested to assess sterility. The results were expressed as the fractional inhibitory concentration index (FICI). The ratio of the MIC of each combination to the MIC of the antimicrobial agent alone was used to determine the drug interaction. The formula for calculating the FICI is shown below:


$$FICI\;=\;\left({FIC}_{A}+\;{FIC}_{B}\right)$$

where:


$$\begin{array}{l} {FIC}_{A}=\frac{\text{MIC of drug A in combination}}{\text{MIC of isolated drug A}}\\ {FIC}_{B}=\frac{\text{MIC of drug B in combination}}{\text{MIC of isolated drug B}} \end{array}$$

The results were interpreted as synergistic interactions (FICI ≤ 0.5), additive effects (0.5 < FICI ≤ 1.0), indifferent effects (1.0 < FICI ≤ 2.0), or antagonistic effects (FICI > 2) (50, 51).

The synergistic interaction between CRO/BLI-489 was also examined using the zero interaction potency (ZIP) model, which was calculated using the free and open-source SynergyFinder software (https://synergyfinder.fimm.fi). The ZIP scores of the combinations were interpreted as follows: synergistic (>10), additive (<10 and >−10), and antagonistic (<−10) (48). Additionally, bacterial growth curves were monitored spectrophotometrically at intervals of 0, 2, 4, 6, 8, 12, and 24 h. The OD was measured at 595 nm using an iMark microplate absorbance reader (Bio-Rad, São Paulo, Brazil) to evaluate the effects of the combination over time (52).

### Antibiofilm formation assay

The ability of the CRO/BLI-489 to inhibit biofilm development was determined using checkerboard test concentrations. Briefly, bacterial suspensions (NDM-*Kp*, 1.5 × 10^6^ CFU/mL) were incubated with CRO and BLI-489 (alone and combined) at 37°C for 24 h under static conditions, allowing biofilm maturation. Next, planktonic cells were removed through three serial washes with sterile water, and the remaining biofilms were stained with 0.1% crystal violet for 30 min, as described (53). The wells were washed again to remove any excess dye, and the surface-bound dye was dissolved in 200 μL of 96% ethanol for 20 min at 4°C to prevent ethanol evaporation. A positive control (NDM-*Kp*) and a sterile control (negative control) were used. The biomass of the biofilm was quantified by measuring the optical density (OD) at 490 nm (54). The percentage of inhibition was calculated using the following formula:


$$\text{Biofilm inhibition}\;(\%)=\frac{{\text{OD}}_{\text{positive control}}-{\text{OD}}_{\text{treatment}}}{{\text{OD}}_{\text{positive control}}}\times 100$$

### Hemolysis assay

Hemolysis assays were performed to analyze the combination hemolytic potential (51).

Briefly, 100 μL of blood erythrocytes was exposed to 100 μL of CRO/BLI-489 at the MIC and MBC, as well as to individual compounds at 64 µg/mL, followed by 4 h of incubation. Next, the samples were centrifuged at 2,500 rpm for 5 min, and the supernatant was transferred to a new microplate. The OD was measured at 595 nm using an iMark microplate absorbance reader (Bio-Rad). Triton X-100 (0.1%, vol/vol) and Dulbecco’s phosphate-buffered saline (D-PBS) were used as positive and negative controls, respectively. The hemolytic rate (HR) was calculated using the following formula:


$$\text{Hemolysis}\;(\%)=\frac{{\text{OD}}_{\text{treatment}}-{\text{OD}}_{\text{negative control}}}{{\text{OD}}_{\text{positive control}}-{\text{OD}}_{\text{negative control}}}\times 100$$

### Cell membrane permeability assay

To determine whether the mechanism of action of the bacteriostatic and bactericidal combinations of CRO/BLI-489 is related to damage to cell membrane integrity, a protein leakage assay was conducted. Briefly, a standardized inoculum of NDM-*Kp* was treated with CRO/BLI-489 and incubated for 4 h at 37°C. After incubation, the samples were centrifuged at 2,500 rpm for 5 min at 4°C. The quantity of protein released from the cytoplasm was determined in the supernatant using a Pierce BCA Protein Assay Kit (Thermo Scientific, MA, USA). OD was measured at 595 nm using an iMark microplate absorbance reader (BioRad, São Paulo, Brazil) (48).

### Scanning electron microscopy (SEM)

Morphological changes in the cellular structure of NDM-*Kp* caused by CRO/BLI489 were analyzed via SEM. Bacteria were incubated in microtubes at 37°C for 24 h. Next, the cultures were washed with phosphate buffer (0.1 M, pH 7.4) and fixed with 2.5% glutaraldehyde. After fixation, the samples were dehydrated with a graded series of ethanol solutions (30, 50, 70, and 100%, vol/vol) for 10 min each. Finally, 20 µL of the final suspension was applied to glass coverslips (0.8 × 0.8 cm) for drying, sputter-coated with gold, and then analyzed via SEM (JSM-6380LV, JEOL, USA).

### Molecular docking and dynamic simulations

For *in silico* investigations, we retrieved the co-crystallized structure of metallo-β-lactamase (MBL; PDB entry: 4EY2) from the Research Collaboratory for Structural Bioinformatics Protein Data Bank (RCSB PDB; https://www.rcsb.org/). This specific PDB entry was selected after the docking protocol was validated, as described in the subsequent sections.

The native conformation of the target protein was initially refined through MD simulations conducted before docking studies. The MBL structure was solvated using the TIP3P water model, and MD simulations were performed using Schrödinger’s Desmond software. These simulations used an orthorhombic simulation box with periodic boundary conditions, ensuring a minimum distance of 10 Å from the protein surface. Before the production phase of the MD simulation, an equilibration step was conducted to stabilize the system. The 150 ns trajectory generated during MD simulations was performed at 300 K and 1.01325 bar pressure. To neutralize the net charge of the system, a 0.15 M NaCl solution was added. The most stable MBL conformation observed after MD simulations was used for subsequent molecular docking studies.

The BLI-489 ligand was sketched and converted into a three-dimensional structure using the MarvinSketch software. Its protonation state at neutral pH (7.4) was assigned, and energy minimization was performed using the ArgusLab v. 4.0.1 software. The docking simulations were performed with the Gold 2020 v.1.10.5 software, which targets the active site of MBL.

The docking protocol was validated by calculating the root mean square deviation (RMSD) obtained from the superposition of the docked ligands and their crystallographic structures from 25 original PDB entries in the RCSB PDB database. This evaluation used all four scoring functions available in Gold 2020: CHEMPLP, GoldScore, ChemScore, and Astex Statistical Potential (ASP). Molecular alignments between co-crystallized and redocked ligands were conducted using the PyMOL v. 2.5 software, and the Chimera software was used to generate 3D illustrations.

The lowest-energy binding poses of BLI-489 were selected as the initial conformation for another 150 ns of MD simulation of the protein-ligand complex. These simulations were performed under the same conditions as those described earlier. Finally, the RMSD was calculated and analyzed using the Maestro graphical interface to support the conclusions drawn (55–57).

### Statistical analysis

All experiments were conducted in triplicate, and the results are expressed as the mean ± standard deviation. Statistical significance between the experimental groups was assessed by conducting one-way analysis of variance (ANOVA), followed by Tukey’s test, with a significance threshold of *P* < 0.01. All data were analyzed using the R programming language, while GraphPad Prism version 10.0.2 was used to generate graphs.

## References

[1] Naghavi M, Vollset SE, Ikuta KS, Swetschinski LR, Gray AP, Wool EE, Robles Aguilar G, Mestrovic T, Smith G, Han C, et al.. 2024. Global burden of bacterial antimicrobial resistance 1990–2021: a systematic analysis with forecasts to 2050. Lancet 404:1199–1226. doi:10.1016/S0140-6736(24)01867-1 39299261 PMC11718157

[2] Sati H, Tacconelli E, Carrara E, Savoldi A, Garcia-Vello P, Zignol M, Cameron A. 2024. WHO bacterial priority pathogens list.

[3] Wise MG, Karlowsky JA, Mohamed N, Hermsen ED, Kamat S, Townsend A, Brink A, Soriano A, Paterson DL, Moore LSP, Sahm DF. 2024. Global trends in carbapenem- and difficult-to-treat-resistance among World Health Organization priority bacterial pathogens: ATLAS surveillance program 2018-2022. J Glob Antimicrob Resist 37:168–175. doi:10.1016/j.jgar.2024.03.020 38608936

[4] Gorrie CL, Mirčeta M, Wick RR, Judd LM, Lam MMC, Gomi R, Abbott IJ, Thomson NR, Strugnell RA, Pratt NF, Garlick JS, Watson KM, Hunter PC, Pilcher DV, McGloughlin SA, Spelman DW, Wyres KL, Jenney AWJ, Holt KE. 2022. Genomic dissection of Klebsiella pneumoniae infections in hospital patients reveals insights into an opportunistic pathogen. Nat Commun 13:3017. doi:10.1038/s41467-022-30717-6 35641522 PMC9156735

[5] Li Y, Kumar S, Zhang L, Wu H, Wu H. 2023. Characteristics of antibiotic resistance mechanisms and genes of Klebsiella pneumoniae. Open Med 18. doi:10.1515/med-2023-0707 PMC10183727 37197355

[6] Al Bshabshe A, Al-Hakami A, Alshehri B, Al-Shahrani KA, Alshehri AA, Al Shahrani MB, Assiry I, Joseph MR, Alkahtani A, Hamid ME. 2020. Rising Klebsiella pneumoniae infections and its expanding drug resistance in the intensive care unit of a tertiary healthcare hospital, Saudi Arabia. Cureus 12:e10060. doi:10.7759/cureus.10060 32999783 PMC7520404

[7] Taha ZM. 2024. Characterization, antibiotic susceptibility, and clonal analysis of carbapenem-resistant Klebsiella pneumoniae from different clinical cases. Cureus 16:e71889. doi:10.7759/cureus.71889 39564009 PMC11573929

[8] Li L, Gao X, Li M, Liu Y, Ma J, Wang X, Yu Z, Cheng W, Zhang W, Sun H, Song X, Wang Z. 2024. Relationship between biofilm formation and antibiotic resistance of Klebsiella pneumoniae and updates on antibiofilm therapeutic strategies. Front Cell Infect Microbiol 14. doi:10.3389/fcimb.2024.1324895 PMC10920351 38465230

[9] Ambler RP. 1980. The structure of beta-lactamases. Philos Trans R Soc Lond B Biol Sci 289:321–331. doi:10.1098/rstb.1980.0049 6109327

[10] Tamma PD, Heil EL, Justo JA, Mathers AJ, Satlin MJ, Bonomo RA. 2024 Infectious diseases society of America 2024 guidance on the treatment of antimicrobial-resistant Gram-negative infections. Clin Infect Dis. doi:10.1093/cid/ciae403 39108079

[11] Harris H, Tao L, Jacobs EB, Bergman Y, Adebayo A, Tekle T, Lewis S, Dahlquist A, Abbey TC, Wenzler E, Humphries R, Simner PJ. 2023. Multicenter evaluation of an mic-based aztreonam and ceftazidime-avibactam broth disk elution test. J Clin Microbiol 61:e01647-22. doi:10.1128/jcm.01647-22 37070979 PMC10204635

[12] Bonomo RA, Burd EM, Conly J, Limbago BM, Poirel L, Segre JA, Westblade LF. 2018. Carbapenemase-producing organisms: a global scourge. Clin Infect Dis 66:1290–1297. doi:10.1093/cid/cix893 29165604 PMC5884739

[13] Heil EL, McCreary EK. 2025. REVISITing treatment of metallo-β-lactamases. Lancet Infect Dis 25:144–146. doi:10.1016/S1473-3099(24)00561-9 39389074

[14] Mendes RE, Doyle TB, Streit JM, Arhin FF, Sader HS, Castanheira M. 2021. Investigation of mechanisms responsible for decreased susceptibility of aztreonam/avibactam activity in clinical isolates of Enterobacterales collected in Europe, Asia and Latin America in 2019. J Antimicrob Chemother 76:2833–2838. doi:10.1093/jac/dkab279 34436603 PMC8561256

[15] Xiang X, Kong J, Zhang J, Zhang X, Qian C, Zhou T, Sun Y. 2025. Multiple mechanisms mediate aztreonam-avibactam resistance in Klebsiella pneumoniae: driven by KPC-2 and OmpK36 mutations. Int J Antimicrob Agents 65:107425. doi:10.1016/j.ijantimicag.2024.107425 39734051

[16] Gales AC, Stone G, Sahm DF, Wise MG, Utt E. 2023. Incidence of ESBLs and carbapenemases among Enterobacterales and carbapenemases in Pseudomonas aeruginosa isolates collected globally: results from ATLAS 2017-2019. J Antimicrob Chemother 78:1606–1615. doi:10.1093/jac/dkad127 37161662 PMC10320057

[17] Liu H, Xiang Y, Xiong M, Xiao X, Zhou J, Tian H, Chen Q, Li Y. 2024. Prevalence of ST1049-KL5 carbapenem-resistant Klebsiella pneumoniae with a bla_KPC-2_ and bla_NDM-1_ co-carrying hypertransmissible IncM1 plasmid. Commun Biol 7:695. doi:10.1038/s42003-024-06398-w 38844513 PMC11156905

[18] Carcione D, Siracusa C, Sulejmani A, Leoni V, Intra J. 2021. Old and new beta-lactamase inhibitors: molecular structure, mechanism of action, and clinical use. Antibiotics (Basel) 10:995. doi:10.3390/antibiotics10080995 34439045 PMC8388860

[19] Venkatesan AM, Agarwal A, Abe T, Ushirogochi H, Yamamura I, Kumagai T, Petersen PJ, Weiss WJ, Lenoy E, Yang Y, Shlaes DM, Ryan JL, Mansour TS. 2004. Novel imidazole substituted 6-methylidene-penems as broad-spectrum β-lactamase inhibitors. Bioorg Med Chem 12:5807–5817. doi:10.1016/j.bmc.2004.08.039 15498657

[20] Venkatesan AM, Agarwal A, Abe T, Ushirogochi H, Yamamura I, Ado M, Tsuyoshi T, Dos Santos O, Gu Y, Sum F-W, Li Z, Francisco G, Lin Y-I, Petersen PJ, Yang Y, Kumagai T, Weiss WJ, Shlaes DM, Knox JR, Mansour TS. 2006. Structure-activity relationship of 6-methylidene penems bearing 6,5 bicyclic heterocycles as broad-spectrum beta-lactamase inhibitors: evidence for 1,4-thiazepine intermediates with C7 R stereochemistry by computational methods. J Med Chem 49:4623–4637. doi:10.1021/jm060021p 16854068

[21] Shi S, Zhang X, Yao Z, Xu M, Zhou B, Liu Q, Zhang Y, Zhou C, Zhou T, Ye J. 2022. Synergistic effect of the novel β-lactamase inhibitor BLI-489 combined with imipenem or meropenem against diverse carbapenemase-producing carbapenem-resistant Enterobacterales. J Antimicrob Chemother 77:1301–1305. doi:10.1093/jac/dkac037 35165715

[22] Petersen PJ, Jones CH, Venkatesan AM, Mansour TS, Projan SJ, Bradford PA. 2009. Establishment of in vitro susceptibility testing methodologies and comparative activities of piperacillin in combination with the penem β-lactamase inhibitor bli-489. Antimicrob Agents Chemother 53:370–384. doi:10.1128/AAC.01047-08 19001109 PMC2630610

[23] Petersen PJ, Jones CH, Venkatesan AM, Bradford PA. 2009. Efficacy of piperacillin combined with the penem β-lactamase inhibitor bli-489 in murine models of systemic infection. Antimicrob Agents Chemother 53:1698–1700. doi:10.1128/AAC.01549-08 19188386 PMC2663108

[24] Ruzin A, Petersen PJ, Jones CH. 2010. Resistance development profiling of piperacillin in combination with the novel {beta}-lactamase inhibitor BLI-489. J Antimicrob Chemother 65:252–257. doi:10.1093/jac/dkp435 20008048

[25] Wang Y-C, Huang S-W, Chiang M-H, Lee I-M, Kuo S-C, Yang Y-S, Chiu C-H, Su Y-S, Chen T-L, Wang F-D, Lee Y-T. 2021. In vitro and in vivo activities of imipenem combined with BLI-489 against class D β-lactamase-producing Acinetobacter baumannii. J Antimicrob Chemother 76:451–459. doi:10.1093/jac/dkaa421 33057603

[26] Mhango M, Sheehan F, Marmor A, Thirkell C, Kennedy K. 2024. An outbreak of double carbapenemase-producing Klebsiella pneumoniae, harbouring ndm-5 and oxa-48 genes, at a tertiary hospital in canberra, Australia. Commun Dis Intell. doi:10.33321/cdi.2024.48.50 39438264

[27] Pawlak M, Lewtak K, Nitsch-Osuch A. 2022. Epidemiology of infections and colonization caused by Klebsiella pneumoniae NDM in the Mazovian Voivodeship in 2016–2017. Postępy Higieny i Medycyny Doświadczalnej 76:275–281. doi:10.2478/ahem-2022-0024

[28] Lutgring JD, Balbuena R, Reese N, Gilbert SE, Ansari U, Bhatnagar A, Boyd S, Campbell D, Cochran J, Haynie J, Ilutsik J, Longo C, Swint S, Rasheed JK, Brown AC, Karlsson M. 2020. Antibiotic susceptibility of NDM-producing Enterobacterales collected in the United States in 2017 and 2018. Antimicrob Agents Chemother 64:e00499-20. doi:10.1128/AAC.00499-20 32540972 PMC7449154

[29] Emeraud C, Mahamat A, Jousset AB, Bernabeu S, Goncalves T, Pommier C, Girlich D, Birer A, Rodriguez C, Pawlotsky J-M, Naas T, Bonnin RA, Dortet L. 2023. Emergence and rapid dissemination of highly resistant NDM-14-producing Klebsiella pneumoniae ST147, France, 2022. Euro Surveill 28:2300095. doi:10.2807/1560-7917.ES.2023.28.42.2300095 37855905 PMC10588306

[30] Sanikhani R, Akbari M, Hosseinzadeh M, Siavash M, Badmasti F, Solgi H. 2024. Outbreak of colistin and carbapenem-resistant Klebsiella pneumoniae ST16 co-producing NDM-1 and OXA-48 isolates in an Iranian hospital. BMC Microbiol 24:59. doi:10.1186/s12866-024-03207-6 38368365 PMC10874040

[31] Zhu J, Wang G, Li M. 2024. Outbreak of NDM-5-producing Klebsiella pneumoniae ST307: an emerging high-risk antimicrobial resistance clone in Shanghai, China. mSystems 9:e01369-23. doi:10.1128/msystems.01369-23 38506533 PMC11019902

[32] Magobo RE, Ismail H, Lowe M, Strasheim W, Mogokotleng R, Perovic O, Kwenda S, Ismail A, Makua M, Bore A, Phayane R, Naidoo H, Dennis T, Ngobese M, Wijnant W, Govender NP, for Baby GERMS-SA1. 2023. Outbreak of NDM-1- and OXA-181-producing Klebsiella pneumoniae bloodstream infections in a neonatal unit, South Africa. Emerg Infect Dis 29:1531–1539. doi:10.3201/eid2908.230484 37486166 PMC10370860

[33] A. Saleh A, A. J. Mahmood A. 2023. Novel β-lactamase inhibitors and the pursuit of MBL inhibitors to combat antibiotic-resistant bacteria: a review. Sci Arch 4:199–207. doi:10.47587/SA.2023.4304

[34] Richards DM, Heel RC, Brogden RN, Speight TM, Avery GS. 1984. Ceftriaxone. Drugs (Abingdon Engl) 27:469–527. doi:10.2165/00003495-198427060-00001 6329638

[35] Kizito M, Lalitha R, Kajumbula H, Ssenyonga R, Muyanja D, Byakika-Kibwika P. 2021. Antibiotic prevalence study and factors influencing prescription of WHO watch category antibiotic ceftriaxone in a tertiary care private not for profit hospital in Uganda. Antibiotics (Basel) 10:1167. doi:10.3390/antibiotics10101167 34680748 PMC8532977

[36] Sonda TB, Horumpende PG, Kumburu HH, van Zwetselaar M, Mshana SE, Alifrangis M, Lund O, Aarestrup FM, Chilongola JO, Mmbaga BT, Kibiki GS. 2019. Ceftriaxone use in a tertiary care hospital in Kilimanjaro, Tanzania: a need for a hospital antibiotic stewardship programme. PLoS One 14:e0220261. doi:10.1371/journal.pone.0220261 31381579 PMC6681960

[37] Tafere C, Endeshaw D, Demsie DG, Yismaw MB, Tefera BB, Yehualaw A, Feyisa K, Siraj EA, Yayehrad AT, Addisu ZD, Adal O. 2024. Inappropriate ceftriaxone utilization and predictor factors in Ethiopia: a systematic review and meta-analysis. Sci Rep 14:25035. doi:10.1038/s41598-024-75728-z 39443593 PMC11499654

[38] Varma A, Warghane A, Dhiman NK, Paserkar N, Upadhye V, Modi A, Saini R. 2023. The role of nanocomposites against biofilm infections in humans. Front Cell Infect Microbiol 13:1104615. doi:10.3389/fcimb.2023.1104615 36926513 PMC10011468

[39] Assefa M, Amare A. 2022. Biofilm-associated multi-drug resistance in hospital-acquired infections: a review. Infect Drug Resist 15:5061–5068. doi:10.2147/IDR.S379502 36068834 PMC9441148

[40] Gao W, Han X, Sun D, Li Y, Liu X, Yang S, Zhou Z, Qi Y, Jiao J, Zhao J. 2023. Antibacterial properties of antimicrobial peptide HHC36 modified polyetheretherketone. Front Microbiol 14:1103956. doi:10.3389/fmicb.2023.1103956 36998411 PMC10043374

[41] Maguigan KL, Al-Shaer MH, Peloquin CA, Maguigan KL, Al-Shaer MH, Peloquin CA. 2021. Beta-lactams dosing in critically ill patients with gram-negative bacterial infections: a PK/PD approach. Antibiotics (Basel) 10:1154. doi:10.3390/antibiotics10101154 34680734 PMC8532626

[42] Jia Y, Schroeder B, Pfeifer Y, Fröhlich C, Deng L, Arkona C, Kuropka B, Sticht J, Ataka K, Bergemann S, Wolber G, Nitsche C, Mielke M, Leiros H-K, Werner G, Rademann J. 2023. Kinetics, thermodynamics, and structural effects of quinoline-2-carboxylates, zinc-binding inhibitors of new delhi metallo-β-lactamase-1 re-sensitizing multidrug-resistant bacteria for carbapenems. J Med Chem 66:11761–11791. doi:10.1021/acs.jmedchem.3c00171 37585683

[43] Denakpo E, Naas T, Iorga BI. 2023. An updated patent review of metallo-β-lactamase inhibitors (2020-2023). Expert Opin Ther Pat 33:523–538. doi:10.1080/13543776.2023.2262763 37737836

[44] Patel S, Jadav P, Bahekar R, Nagaswamy K, Viswanathan K, Vyas P, Giri P, Sachchidanand S, Jain M. 2024. Design and biological evaluation of cephalosporin based metallo-β-lactamase (MBL) inhibitors. LDDD 21:3506–3514. doi:10.2174/0115701808287192240415062148

[45] Isler B, Aslan AT, Akova M, Harris P, Paterson DL. 2022. Treatment strategies for OXA-48-like and NDM producing Klebsiella pneumoniae infections. Expert Rev Anti Infect Ther 20:1389–1400. doi:10.1080/14787210.2022.2128764 36150216

[46] Vaz MSM, de Almeida de Souza GH, Dos Santos Radai JA, Fraga TL, de Oliveira GG, Wender H, da Silva KE, Simionatto S. 2023. Antimicrobial activity of cinnamaldehyde against multidrug-resistant Klebsiella pneumoniae: an in vitro and in vivo study. Braz J Microbiol 54:1655–1664. doi:10.1007/s42770-023-01040-z 37392293 PMC10485196

[47] CLSI. 2015. M100-S25 performance standards for antimicrobial susceptibility testing; twenty-fifth informational supplement. Clinical and Laboratory Standards Institute (CLSI), Wayne, PA.

[48] Vasconcelos NG, Queiroz JHF de S, Silva KE da, Vasconcelos PC de P, Croda J, Simionatto S. 2020. Synergistic effects of Cinnamomum cassia L. essential oil in combination with polymyxin B against carbapenemase-producing Klebsiella pneumoniae and Serratia marcescens. PLoS One 15:e0236505. doi:10.1371/journal.pone.0236505 32701970 PMC7377461

[49] Surana A, Priya C, Bhavya A, Suparna GS, Rolly SA, Kewlani M. 2024. Comparative evaluation of minimal inhibitory concentration and minimal bactericidal concentration of various herbal irrigants against Enterococcus faecalis. J Conserv Dent Endod 27:780–784. doi:10.4103/JCDE.JCDE_349_23 39262589 PMC11385917

[50] Zhang W, Guo Y, Li J, Zhang Y, Yang Y, Dong D, Zhu D, He P, Hu F. 2018. In vitro and in vivo bactericidal activity of ceftazidime-avibactam against Carbapenemase–producing Klebsiella pneumoniae. Antimicrob Resist Infect Control 7:142. doi:10.1186/s13756-018-0435-9 30479755 PMC6249859

[51] Sturaro MC, de Souza GH de A, Damaceno N da S, Silva ON, de Aquino TM, Freire NML, Alcântara MGDS, Monteiro KLC, Martins AA, Rossato L, Fraga TL, Borsuk S, Dellagostin OA, Simionatto S. 2025. Antimicrobial activity of ceftibuten/polymyxin B combination against polymyxin/carbapenem-resistant Klebsiella pneumoniae. J Antimicrob Chemother 80:116–125. doi:10.1093/jac/dkae382 39450857

[52] Wang J, Ma X, Li J, Shi L, Liu L, Hou X, Jiang S, Li P, Lv J, Han L, Cheng Y, Han B. 2023. The synergistic antimicrobial effect and mechanism of nisin and oxacillin against methicillin-resistant Staphylococcus aureus. IJMS 24:6697. doi:10.3390/ijms24076697 37047670 PMC10094802

[53] Ribeiro SM, de la Fuente-Núñez C, Baquir B, Faria-Junior C, Franco OL, Hancock REW. 2015. Antibiofilm peptides increase the susceptibility of carbapenemase-producing Klebsiella pneumoniae clinical isolates to β-lactam antibiotics. Antimicrob Agents Chemother 59:3906–3912. doi:10.1128/AAC.00092-15 25896694 PMC4468710

[54] Rossato L, Arantes JP, Ribeiro SM, Simionatto S. 2022. Antibacterial activity of gallium nitrate against polymyxin-resistant Klebsiella pneumoniae strains. Diagn Microbiol Infect Dis 102:115569. doi:10.1016/j.diagmicrobio.2021.115569 34775292

[55] Viana LPS, Naves GM, Medeiros IG, Guimarães AS, Sousa ES, Santos JCC, Freire NML, de Aquino TM, Modolo LV, de Fátima Â, da Silva CM. 2024. Synergizing structure and function: cinnamoyl hydroxamic acids as potent urease inhibitors. Bioorg Chem 146:107247. doi:10.1016/j.bioorg.2024.107247 38493635

[56] Nunes JA, Araújo RSA de, Silva FN da, Cytarska J, Łączkowski KZ, Cardoso SH, Mendonça-Júnior FJB, Silva-Júnior EF da. 2023. Coumarin-based compounds as inhibitors of tyrosinase/tyrosine hydroxylase: synthesis, kinetic studies, and in silico approaches. Int J Mol Sci 24:5216. doi:10.3390/ijms24065216 36982292 PMC10048804

[57] da Silva AC, Marques AM, Figueiredo MR, de Aguiar JCR de OF, da Câmara CAG, de Moraes MM, de Oliveira APS, Napoleão TH, Paiva PMG, de Aquino TM, da Silva-Júnior EF, Crotti AEM, Navarro DM do AF. 2023. Larvicidal activity, enzyme inhibitory effect, and molecular docking by essential oil, hydrolate, aqueous extract, and major compounds from the leaves of Eugenia uniflora against Aedes aegypti. Ind Crops Prod 204:117380. doi:10.1016/j.indcrop.2023.117380

